# Impact of video quality when evaluating video-assisted cardiopulmonary resuscitation: a randomized, controlled simulation trial

**DOI:** 10.1186/s12873-021-00486-4

**Published:** 2021-08-21

**Authors:** Christopher Plata, Martin Nellessen, Rebecca Roth, Hannes Ecker, Bernd W. Böttiger, Johannes Löser, Wolfgang A. Wetsch

**Affiliations:** 1 University of Cologne, Faculty of Medicine and University Hospital of Cologne, Department of Anaesthesiology and Intensive Care Medicine, Kerpener Strasse 62, 50937 Cologne, Germany; 2 Emergency Department, University Hospital RWTH Aachen, Pauwelsstrasse 30, 52074 Aachen, Germany; 3 University of Cologne, Faculty of Medicine, and University Hospital of Cologne, Institute of Medical Statistics and Computational Biology, Kerpener Strasse 62, 50937 Cologne, Germany; 4 University of Cologne, Faculty of Medicine and University Hospital of Cologne, Centre of Palliative Medicine, Kerpener Strasse 62, 50937 Cologne, Germany

**Keywords:** Cardiopulmonary resuscitation, CPR, Video-assisted CPR, V-CPR, OHCA

## Abstract

**Background:**

Although not routinely established during cardiopulmonary resuscitation (CPR), video-assisted CPR has been described as beneficial in the communication with emergency medical service (EMS) authorities in out-of-hospital cardiac arrest scenarios. Since the influence of video quality has not been investigated systematically and due to variation of quality of a live-stream video during video-assisted CPR, we investigated the influence of different video quality levels during the evaluation of CPR performance in video sequences.

**Methods:**

Seven video sequences of CPR performance were recorded in high quality and artificially reduced to medium and low quality afterwards. Video sequences showed either correct CPR performance or one of six typical errors: too low and too high compression rate, superficial and increased compression depth, wrong hand position and incomplete release. Video sequences were randomly assigned to the different quality levels. During the randomised and double-blinded evaluation process, 46 paramedics and 47 emergency physicians evaluated seven video sequences of CPR performance in different quality levels (high, medium and low resolution).

**Results:**

Of 650 video sequences, CPR performance was evaluable in 98.2%. CPR performance was correctly evaluated in 71.5% at low quality, in 76.8% at medium quality, and in 77.3% at high quality level, showing no significant differences depending on video quality (*p* = 0.306). In the subgroup analysis, correct classification of increased compression depth showed significant differences depending on video quality (*p* = 0.006). Further, there were significant differences in correct CPR classification depending on the presented error (*p* < 0.001). Allegedly errors, that were not shown in the video sequence, were classified in 28.3%, insignificantly depending on video quality. Correct evaluation did not show significant interprofessional differences (*p* = 0.468).

**Conclusion:**

Video quality has no significant impact on the evaluation of CPR in a video sequence. Even low video quality leads to an acceptable rate of correct evaluation of CPR performance. There is a significant difference in evaluation of CPR performance depending on the presented error in a video sequence.

**Trial registration:**

German Clinical Trial Register (Registration number DRKS00015297) Registered on 2018-08-21.

## Background

Sudden cardiac arrest is the most immediate life-threatening medical condition. Despite all advances in prevention and treatment, it accounts for approximately half of all deaths from cardiovascular disease [1], and is hence among the leading causes of death in industrialised nations [2]. Brain tissue is very vulnerable to hypoxaemia, and irreversible damage starts to occur 3 to 5 min after the onset of circulatory arrest. Unfortunately, despite rapid response by Emergency Medical Services (EMS), professional helpers often arrive too late on scene to save patients’ lives or avoid irreversal neurological damage. Early initiation of cardiopulmonary resuscitation (CPR) by bystanders can bridge the gap till the arrival of medical professionals and hence could help save hundreds of lives every day [3, 4]. Sadly, in most countries, bystander CPR in out-of-hospital cardiac arrest is performed in only 15–50% [5], although witnessed in more than 60% of the cases [2, 6].

By introducing dispatcher-assisted telephone CPR (T-CPR), which contains structured instruction on how to perform CPR, the probability of surviving cardiac arrest has considerably increased [4, 7–10]. However, instructing bystanders, who are mostly unexperienced in providing CPR, remains challenging. Due to the limitations of the audio-only communication, dispatchers cannot see what the bystander does, and hence cannot evaluate CPR quality or give corrective feedback.

Despite technical inventions and revolutionary advantages over the last decades, like the ubiquitous coverage of high-speed internet connectivity and mobile multifunctional smartphones, the predominant way of communication with emergency authorities has remained the traditional audio contact. Video-assisted CPR (V-CPR) is a new possible way of instructing bystanders during CPR [11, 12]. Although first studies of video-assisted CPR started over a decade ago, video-guided CPR did not prove to be superior to conventional CPR at those days. To date, video-assisted CPR remains almost unknown in EMS systems around the world. Recently, this feature has been implemented in a software that can be routinely used for EMS dispatch centres, and first studies have shown that this technology can be used to judge a layperson’s CPR efforts and correct them, if necessary [13].

However, to date there is no priority for mobile data used for emergency calls, hence livestream video quality can vary due to reduced data transfer rates or bad mobile internet coverage. The necessary quality of a video in order to allow accurate judgement of CPR efforts has not been studied so far. Furthermore, in published research, there is heterogeneity concerning the qualification of the evaluating person.

The aim of this study was to determine the necessary video quality in order to be able to evaluate CPR quality. In addition, we wanted to investigate whether there is an influence of the evaluator’s profession on evaluation of CPR quality in a video sequence.

## Methods

### Study design

This prospective, randomized, controlled, parallel group and double-blind simulation trial was conducted in the University Hospital of Cologne and the EMS Department of the Fire Brigade of Kerpen, Germany. The study was approved by the Ethics Committee of the University Hospital Cologne (Approval number 18–130, 12th of February 2019) and was registered at the German Clinical Trial Register (Registration number DRKS00015297). Data was collected from September 2019 to February 2020. Written and informed consent was obtained from each participant prior to inclusion.

### Materials

CPR was simulated using a standardized CPR training manikin (Ambu Man basic, Ambu GmbH, Bad Nauheim, Germany). Video sequences were recorded with a Tamron objective (Tamron SP 24-70 mm F/2.8, Tamron Corporation, Saitama, Japan) on a Nikon D750 (Nikon Corporation, Tokyo, Japan) with a resolution of 1920 × 1080 pixels per inch and 30 frames per second, to obtain a video with maximum quality. The camera was placed laterally of the manikin on a tripod of 1.80 m height, facing the manikin and the helper performing CPR from approximately 2.5 m of distance. A metronome (metronom beats app, Stonekick, London, UK; downloaded from Google Play Store) was set to 110 bpm (or the corresponding lower/higher frequency) to ensure guideline-conformant compression frequency, and the internal compression depth indicator of the manikin was used to ensure sufficient compression depth. In the final video sequences, neither metronome nor compression depth indicator was visible respectively audible. Seven high quality video clips of 12 s each were recorded, showing either guideline-conformant optimum performance compression-only CPR, or a typical error often made during CPR (Table 1).

**Table 1:** CPR parameters and errors shown in the video sequences

1	Correct CPR	Compression depth 5-6 cm, compression rate 110/min, correct hand position, complete thorax release after compression
2	Low compression rate	80 min^−1^, others correct
3	High compression rate	140 min^−1^, others correct
4	Increased compression depth	> 70 mm, others correct
5	Superficial compression depth	30-40 mm, others correct
6	Wrong hand position	Epigastric compression point, others correct
7	Incomplete thorax release	Remaining depth > 20 mm, others correct

**Fig. 1:**
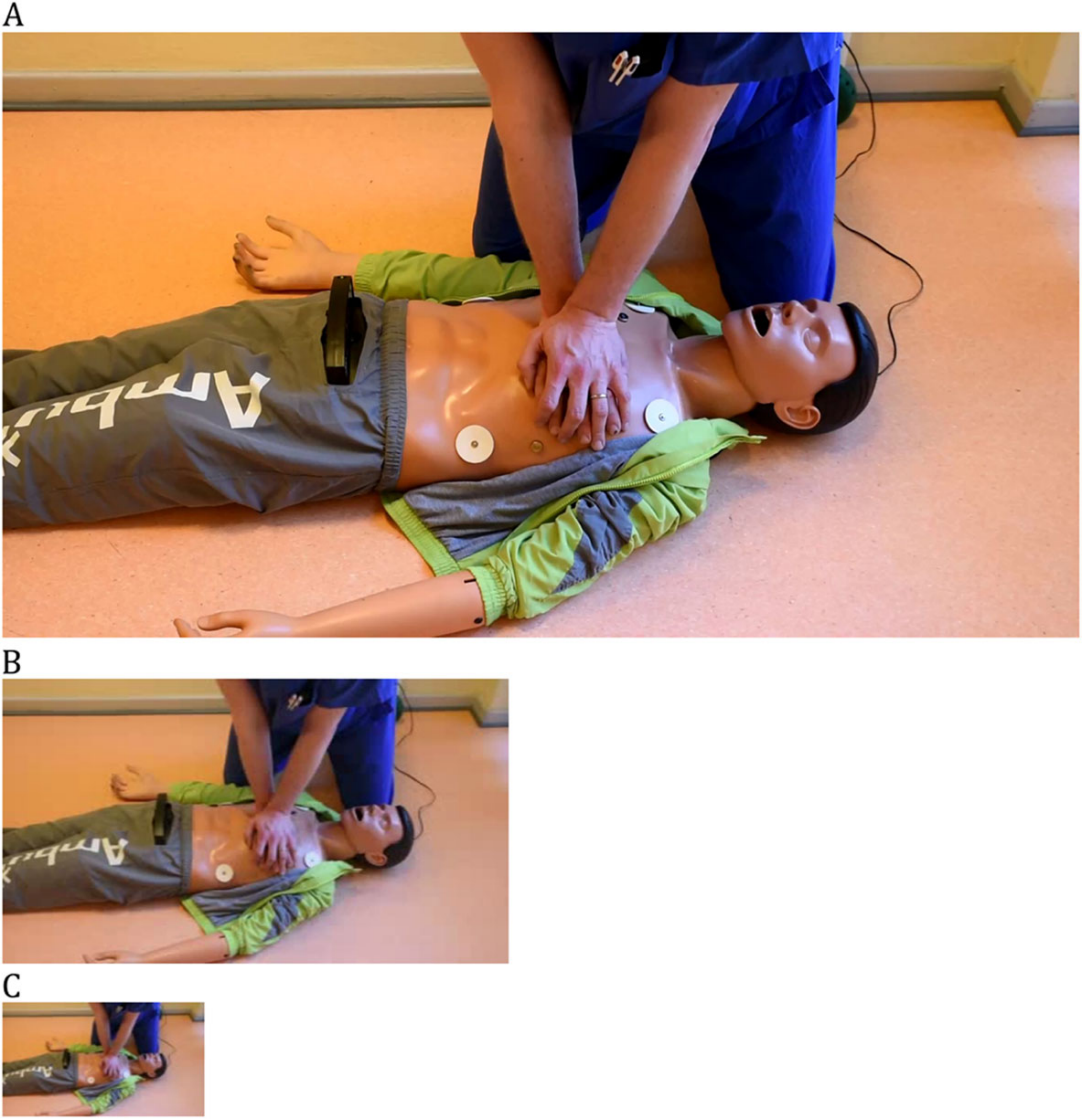
Still frames of the videos used for evaluation: **A** high resolution (1920x1080ppi), **B** medium resolution (320x240ppi) and **C** low resolution (128x96ppi)

Video resolution was artificially reduced by using a software tool (XMedia Recode, V. 3.4.3.6, Eschenbergen, Germany) to obtain three quality levels: high (resolution 1920x1080ppi), medium (resolution 320x240ppi) and low (resolution 128x96ppi), all with a frame rate of 30fps (Fig. 1). High quality served as standard, while the lower resolutions are typically used on mobile devices, when data is transferred via wireless networks and compression is necessary. Video sequences and quality levels were randomized with a randomization algorithm provided by the Institute of Medical Statistics and Computational Biology (IMSB) of the University of Cologne. During the evaluation phase, video sequences were analyzed on an ASUS X541UA laptop with a 15.6″ screen. A standardized questionnaire with multiple-choice answers was used to evaluate CPR quality.

### Study protocol

Foty-six paramedics and 47 emergency physicians were randomly recruited on the campus of the University Hospital of Cologne, Germany and the EMS Department of the Fire Brigade of Kerpen, Germany. There were no primary exclusion criteria.

**Fig. 2:**
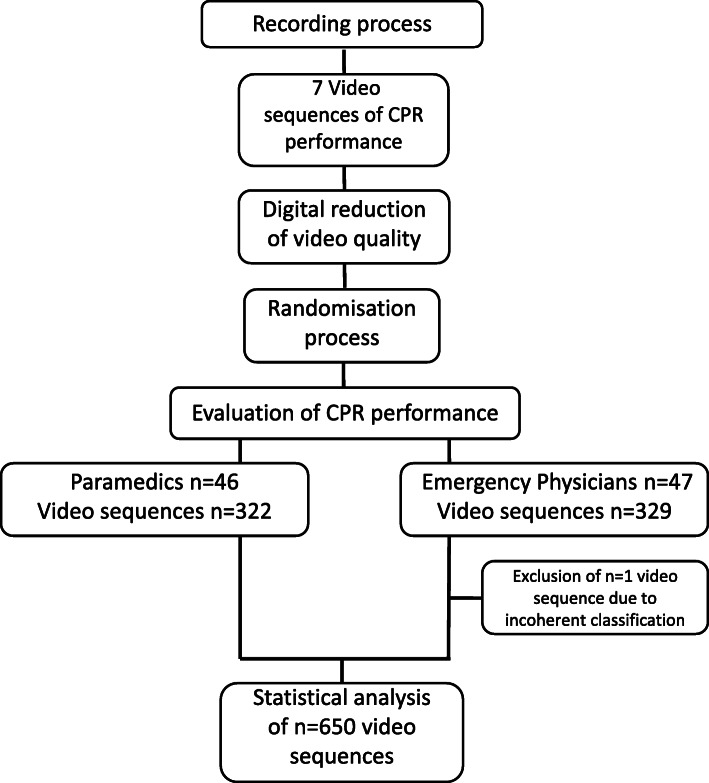
Study flow chart. During the recording process, seven video sequences of CPR were recorded. After reduction of the video quality, video sequences were randomly assigned to the different quality levels and evaluated by either paramedics or emergency physicians, CPR: cardiopulmonary resuscitation

Each evaluator had to judge seven video sequences (Fig. 2). Six of those included the six different error types and one did not comprise any error. Only one error was included per video sequence (see Table 1). Participants were asked to evaluate CPR performance after each video sequence, using a standardized questionnaire with multiple-choice answers about compression rate, compression depth, compression point and release after compression. Additionally, participants were not informed about the number of errors in one video sequence and, thus, indication of more than one error was possible. During the evaluation of every video sequence, participantshad the possibility to indicate each category as “not evaluable”. There was no time limit for answering the questionnaire.

### Measurements and outcomes

The primary outcome parameter was correct identification of the error presented in the video sequence depending on the video quality. Secondary outcome parameters consisted of classification of CPR performance depending on the presented error, false classification of correct CPR performance, frequencies of “CPR performance not evaluable”, indication of further, allegedly errors and finally differences in classification depending on different professions.

### Statistical analysis

The required sample size was calculated in order to achieve 80% power at a significance level of 5% to detect a minimal difference in the percentage of error recognition between two different qualities of 25%, i.e. assuming a detection rate of 95 vs. 70%. Sample size calculation was accounted for a) the dependency of the seven ratings conducted by the same evaluator by assuming an intraclass correlation coefficient of 0.8 and b) a drop-out rate of 10%. When recruiting a total of 100 evaluators, the actual power of a chi-square test to detect a difference in proportions of 25% was estimated as 81% (SAS 9.3, Cary, North Carolina, USA). For each evaluator separately, error types and qualities were randomly assigned to video sequences, such that each evaluator had to judge each quality at least twice. For random assignment of error types and qualities, the software “R” (The R Foundation for Statistical Computing. Vienna, Austria) was employed. Descriptive analyses present numbers and percentages for categorical variables. For statistical analyses, categories out of the seven CPR performances were formed: low and high compression rate were summarized in the category “compression rate”, low and increased compression depth in the category “compression depth” and finally hand position and thorax release. Where exact Fisher’s test could not be calculated due to limited computational capacities, Chi-square tests were applied to investigate associations between categorical variables. Missing data were not imputed. As this is an exploratory study, no correction for multiple testing was performed. *P* values < 0.05 were regarded as statistically significant. Statistical computations were carried out using IBM SPSS Statistics version 25.

## Results

In total, 651 video sequences were presented to the evaluators, including 322 video sequences being evaluated by 46 paramedics and 329 video sequences analyzed by 47 emergency physicians (Fig. 2). All questionnaires were returned. One volunteer of the emergency physicians-group gave inconsistent answers regarding release after compression in a video sequence of medium quality, showing correct CPR performance. Thus, 650 answers (99.8%) were included for statistical analysis.

### Classification of CPR performance depending on video quality

**Fig. 3:**
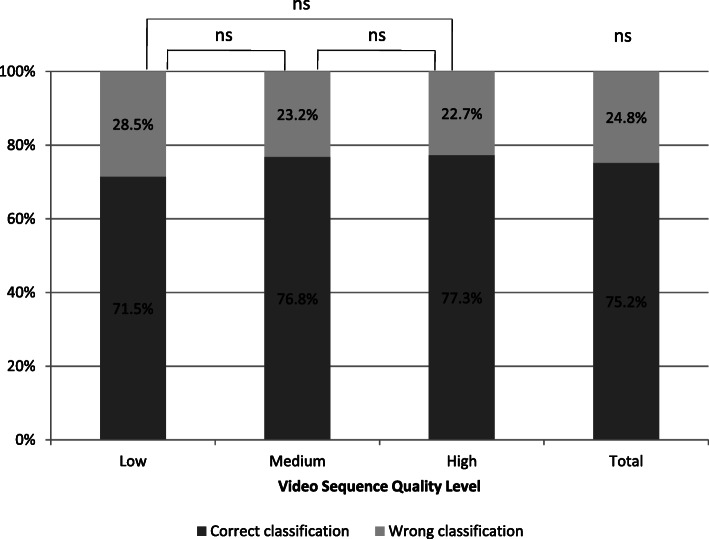
Classification of CPR performance in video sequences of different quality levels; not significant (ns)

Errors presented in the video sequences were evaluated correctly in 71.5% at low quality, in 76.8% at medium quality and in 77.3% at high quality (Fig. 3). Overall, 75.2% of all presented video sequences were correctly evaluated. There was no significant difference in correct error classification depending on quality levels of the presented video sequences (*p* = 0.306).

**Fig. 4:**
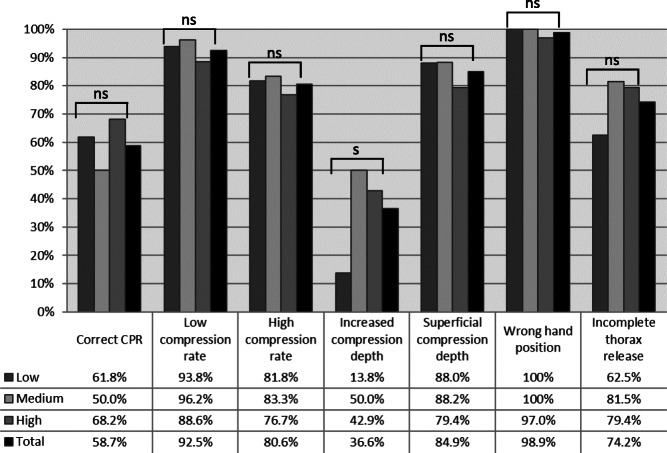
Correct classification of errors during CPR depending on different video quality levels; not significant (ns), significant (s)

Regarding the classification of the specific errors depending on the different video quality levels, results are shown in Fig. 4. Wrong hand position was recognized correctly in 100% at low and medium quality levels and in 97% at high quality level, revealing no significant differences in dependence of video quality (*p* = 1.0). Likewise, correct CPR performance (*p* = 0.363), low and high compression rate (*p* = 0.617; *p* = 0.847), superficial compression depth (*p* = 0.619) and incomplete release (*p* = 0.198) did not show significant differences depending on video quality. In contrast, 13.8% of increased chest compressions at low quality, 50% at medium quality and 42.9% at high quality level were classified correctly, revealing a significant difference in different video quality stages (*p* = 0.006).

### Classification of CPR performance depending on the presented error

**Fig. 5:**
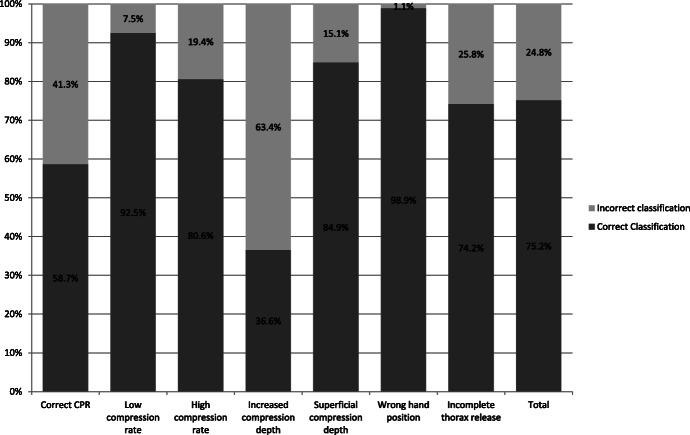
Frequencies of correct error classification depending on the presented error over all quality stages (*p* < 0.001)

Further analyses focussed on the frequencies of correct error classification depending on the presented error. Thereby, we found a significant difference in correct classification depending on the presented error (*p* < 0.001; Fig. 5). In the following subgroup analysis, we evaluated differences in correct classification between two errors, as shown in Table 2.

**Table 2:** Pairwise analysis of CPR performances and levels of significance

CPR performance		CPR performance	***p***-value
Correct CPR	vs	Low compression rate	< 0,001
Correct CPR	vs	High compression rate	0,001
Correct CPR	vs	Increased compression depth	0,003
Correct CPR	vs	Superficial compression depth	< 0,001
Correct CPR	vs	Wrong hand position	< 0,001
Correct CPR	vs	Incomplete thorax release	0,03
Low compression rate	vs	High compression rate	0,03
Low compression rate	vs	Increased compression depth	< 0,001
Low compression rate	vs	Superficial compression depth	0,163
Low compression rate	vs	Wrong hand position	0,064
Low compression rate	vs	Incomplete thorax release	0,001
High compression rate	vs	Increased compression depth	< 0,001
High compression rate	vs	Superficial compression depth	0,561
High compression rate	vs	Wrong hand position	< 0,001
High compression rate	vs	Incomplete thorax release	0,381
Increased compression depth	vs	Superficial compression depth	< 0,001
Increased compression depth	vs	Wrong hand position	< 0,001
Increased compression depth	vs	Incomplete thorax release	< 0,001
Superficial compression depth	vs	Wrong hand position	0,001
Superficial compression depth	vs	Incomplete thorax release	0,101
Wrong hand position	vs	Incomplete thorax release	< 0,001

Correct CPR was falsely evaluated as “incorrect” in 41.3% of all videos and thus evaluators attributed errors to the correct CPR performance. Correct CPR performance was falsely classified as low compression rate (21.6%, [*n* = 8]), superficial compression depth and incomplete thorax release (each 27%, [*n* = 10]), high compression rate, increased compression depth and wrong hand position (each 8.1%, [*n* = 3]).

### CPR performance not evaluable

In 12 of 650 video sequences (1.8%), a CPR category (compression rate, compression depth, hand position, thorax release) was marked as “not evaluable”. In case of low video quality, evaluators indicated a CPR category to be not evaluable in 6 video sequences (0.9%), 1 video sequence (0.2%) in a medium quality level and in 5 high quality video sequences (0.8%), revealing no significant difference depending on video quality level (*p* = 1.0).

### Indication of further, allegedly errors

**Fig. 6:**
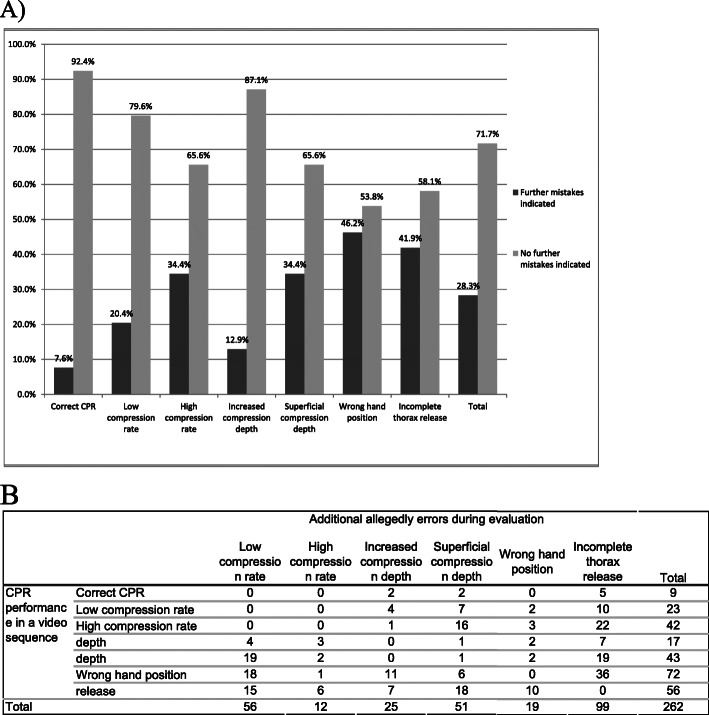
**A** Frequencies of additional errors depending on the presented CPR performance in a video sequence (*p* < 0.001); **B** crosstabulation showing allocation and summation of all additional allegedly errors during evaluation of a video sequence (indication of more than one error was possible)

As shown in Table 1, there was only one error per video sequence (except for the video with correct CPR, which contained no error). However, in 184 of 650 video sequences (28.3%), 262 additional errors, that were not shown in the video sequence, were indicated by the evaluators (Fig. 6b). While allegedly errors did not significantly depend on video quality levels (*p* = 0.422), the presented error was of significance for the indication of additional errors (*p* < 0.001). Lowest frequency of additional allegedly errors was found in video sequences of correct CPR performance (7 of 92 = 7.6%, Fig. 6a). Highest number of indicated additional allegedly errors was seen in video sequences showing wrong hand position during CPR (43 of 93 = 46.2%), revealing a significant difference compared to correct CPR (*p* < 0.001).

### Error classification by different professionals

This study was not powered to find differences in the evaluation of different professions. However, our results reveal some interesting findings regarding interprofessional differences between paramedics and emergency physicians. Summarizing all quality stages, correct classification of the presented errors was achieved in 73.9% for paramedics and in 76.5% for emergency physicians, showing no significant interprofessional difference (*p* = 0.468). Significant interprofessional differences were only seen in low quality video sequences, where 78.7% (*n* = 85) of the presented errors were correctly evaluated by emergency physicians, but only 64.2% (*n* = 68) by paramedics (*p* = 0.023). In medium and high quality, interprofessional evaluation showed no significant differences (*p* = 0.873 and *p* = 0.419 respectively). However, by analysing the results of emergency physicians and paramedics separately, there was a significant influence of the video quality level on correct errors classification by paramedics (*p* = 0.021) but not by emergency physicians (p = 0.8). For paramedics evaluating a video sequence, increased chest compressions in a low video quality were significantly less frequently recognized, compared to medium and high quality levels (12.5% vs. 60% vs. 60%; *p* = 0.007). Further, video quality had a significant influence on detection of incomplete thorax release after compression evaluated by paramedics (*p* = 0.045) but not by emergency physicians (*p* = 0.393). It was most frequently detected at a high quality level (92.3%), followed by a medium quality level (73.3%) and low quality level (50%). In both professional groups, the presented error in a video sequence had a significant influence on correct error classification (p_paramedics_ < 0.001; p_emergency physicians_ < 0.001). Finally, there was no interprofessional difference regarding indicating “CPR performance not evaluable” (*p* = 0.415) and indicating further errors (*p* = 0.728).

## Discussion

The present study is the first to primarily focus on the influence of the video quality on the assessment of CPR effort during simulated V-CPR. Our results indicate that the assessment of CPR quality during video-assisted CPR does not depend on the availability of high-quality video footage, since correct classification did not differ significantly by video quality.

Tremendous developments in technology have brought significant changes to daily life in regard to telecommunication over the last decades [14]. Most visibly during the 2020 SARS-Cov2 pandemic, telemedicine got a major boost and video-connections for doctor’s appointments and hospital triage got more common [15, 16, 17]. Surprisingly, communication with emergency authorities – which is one of the most crucial communication imaginable – seems to be unaffected by these trends and remains a traditional “audio-only” phone call. So far, to our best knowledge, video-assisted CPR has only been adopted and is commercially available in very few regions [13, 18, 19]. Nevertheless, studies have demonstrated that video calls can provide additional information to improve emergency communication, especially in conjunction with CPR [11–13, 18–22].

However, comparing these studies is challenging, as they were mostly not standardized in regard to image quality used and often do not state any quality preferences at all. Further, the term “video-CPR” is not clearly defined and thus comprises not only video-assisted CPR. In a study by Bolle et al., conducted over a decade ago, the authors compared T-CPR and V-CPR with high school students using a Nokia N90 [20]. No superiority of the V-CPR group was shown, which was explained by the low video quality (video resolution 352 × 288 pixels, framerate 15/s), leading to difficulties in identifying the details of CPR performance. Technical conditions in this study are comparable to our medium quality, however we used a framerate of 30/s which is more comparable to the state of the art of video-streaming. Another study by Stipulante et al. compared V-CPR to T-CPR on mobile phones with 180 students [23]. The authors showed that the use of a V-CPR protocol allowed the CPR to reach compression rates and depths close to guidelines and to reduce ‘hands-off’ events. However, device and image quality parameters were not mentioned. More recently, in two of our own previous studies, we tested V-CPR via smartphones in a randomized controlled trial on 150 participants in a wifi-setting and under realistic conditions in a metropolitan area using a mobile telephone network [13, 18]. Both studies used a video livestream from a Samsung Galaxy S7, which delivers a video quality comparable to the medium and high quality setting in the present study. However, video quality was not explicitly stated in these studies. Despite results showing an overall improvement of the CPR quality, authors emphasized that connection problems due to technical issues and signal degradation affected video quality, especially in the second study using the mobile internet coverage.

All of our video sequences were taped with a framerate of 30 fps, as lower frame rates may contribute to an aliasing phenomenon. We consciously did not evaluate video sequences with reduced framerates, as it is very unlikely that they will play an important part in the future with the introduction of 4G and 5G mobile data networks. Furthermore, it is quite possible that higher resolutions and framerates will be available also on mobile devices, as 4 k resolution (4096 × 2160 pixels) and framerates up to 60fps become available, albeit not routinely implemented in video calls yet. Although this study did not show appropriate level of statistical power and sensitivity in regard to the interprofessional differences, results do not show a significant interprofessional difference in the evaluation of V-CPR. It can be carefully suggested that a variety of medical emergency professions can serve as dispatchers for V-CPR, since both professional groups identified over 70% of the CPR performances correctly. Furthermore, it must be kept in mind that our evaluators were confronted with video-assisted CPR for the very first time. None of them had any training with this technology. It can be hypothesized that any training may lead to further improved evaluation of V-CPR [23].

Furthermore, data in both simulated and actual patients have shown that even experienced clinicians are not very good at determining whether compressions are being one properly – this applies for “remote” observers as well as for those that are physically present at the time [24–27]. Judging other persons performing CPR is not taught in any course, and it seems as if those who need to judge other persons’ CPR performance (e.g., 911 dispatchers or telemedicine paramaedics/doctors) might need special training towards evaluation of CPR quality. Further studies evaluating this finding are needed to gain insight into this phenomenon.

### Limitations

This study was conducted with a resuscitation mannequin made for education and evaluation of medical personal, which is only a substitute for real patients. In accordance with the current European Resuscitation Council (ERC) Guidelines [28], we confined to a compression-only CPR approach. Future studies might incorporate ventilation in their CPR scenario. This study only investigated a maximum resolution of 1920 × 1080 pixels and framerate of 30fps. It is quite possible that a higher resolution and framerate can lead to different results as 4 k resolution (4096 × 2160 pixels) and framerates till 60fps are available. Videotaping was performed in a closed environment with sufficient light, constant weather, no disturbances and perfect technical facilities (e.g. tripod). Further evaluation in real-life situations is recommended. It must also be considered that we only used one video position. Different positions may positively or negatively influence the ability to recognize and correct certain mistakes.

## Conclusion

Results of this study show that video quality has no significant impact on the evaluation of CPR in a video sequence. In fact, even low video quality leads to an acceptable evaluation of CPR performance. Except of the parameter “increased compression depth”, not video quality but the presented error was of significance for correct CPR classification.

## Abbreviations

ALS: Advanced Life Support
CPR: Cardio-pulmonary resuscitation
EMS: Emergency medical services
ERC: European Resuscitation Council
GRC: German Resuscitation Council
IBM: International Business Machines Corporation
ILCOR: International Liaison Committee on Resuscitation
IMSB: Institute of Medical Statistics and Computational Biology
OHCA: Out-of-hospital cardiac arrest
SARS-CoV2: severe acute respiratory syndrome coronavirus type 2
SAS: Statistical Analysis Systems
SPSS: Statistical Package for the Social Sciences
T-CPR: Telephone-assisted cardio-pulmonary resuscitation
V-CPR: Video-assisted cardio-pulmonary resuscitation
